# Expired Patents

**Published:** 1906-10

**Authors:** 


### EXPIRED PATENTS.

August 27, 1906.

409,671, Artificial tooth crown, S. B. Dewey, Cleveland, Ohio.

410,083, Dental bib clamp, J. D. Ennes, Norfolk, Va.

Sept. 3, 1906.

410,158, Dental instrument, R. B. Donaldson, Washington, D. C.

Sept. 10, 1906.

410,665, Dental jaw prop, R. H. Daintree, New York, N. Y.

410,674, Dentist's chair, C. Rauhe, Dusseldorf, Germany.

410,935, Dental plugger, F. Schmidt, Demmin, Prussia, Germany.

Sept. 17, 1906.

411,272, Fastening artificial teeth, G. M. Weirich, Phila., Pa.

The above are furnished by Davis & Davis, solicitors of American and foreign patents, Washington, D. C., and St. Paul building, New York City.

